# The Association Between Sleep Disorders and Incidence of Dry Eye Disease in Ningbo: Data From an Integrated Health Care Network

**DOI:** 10.3389/fmed.2022.832851

**Published:** 2022-02-04

**Authors:** Qinxiang Zheng, Saiqing Li, Feng Wen, Zhong Lin, Kemi Feng, Yexiang Sun, Jie Bao, Hongfei Weng, Peng Shen, Hongbo Lin, Wei Chen

**Affiliations:** ^1^The Affiliated Ningbo Eye Hospital of Wenzhou Medical University, Ningbo, China; ^2^Eye Hospital and School of Ophthalmology and Optometry, Wenzhou Medical University, Wenzhou, China; ^3^Department of Chronic Diseases and Health Promotion, Yinzhou District Center for Disease Control and Prevention, Ningbo, China

**Keywords:** sleep disorders, dry eye disease, risk factor, sleep medicine, coexisting disease

## Abstract

**Purpose:**

To investigate the association between sleep disorders and dry eye disease (DED) in Ningbo, China.

**Methods:**

Our data came from the Yinzhou Health Information System (HIS), including 257932 patients and was based on a 1:1 matching method (sleep disorder patients vs. patients without sleep disorders) during 2013–2020. Sleep disorders and DED were identified using ICD-10 codes. Cox proportional hazards regression was used to identify the association between sleep disorders and DED.

**Results:**

The eight-year incidence of DED was significantly higher in participants with diagnosis of sleep disorders (sleep disorders: 50.66%, no sleep disorders: 16.48%, *P* < 0.01). Sleep disorders were positively associated with the diagnosis of DED (HR: 3.06, 95% CI: 2.99–3.13, *P* < 0.01), when sex, age, hypertension, diabetes and other systemic diseases were adjusted. In the sleep disorders patients, advancing age, female sex, and presence of coexisting disease (hypertension, diabetes, hyperlipidemia, thyroid disease, depression, heart disease, and arthritis) were significantly associated with the development of DED by the multivariate cox regression analysis (all *P* < 0.05).In addition, there was a significantly positive association between estazolam and the incidence of DED in both sleep disorder and non-sleep disorder groups (all *P* < 0.05).

**Conclusions:**

Sleep disrder was associated with a three-time increased risk of DED. This association can be helpful in effective management of both sleep disorders and DED.

## Introduction

Sleep disorder, defined as an abnormality in sleep quality or quantity, has become a major social health concern throughout the world (1–3). Sleep quality and duration of sleep are important for physical and mental health, and sleep dysfunction damages autonomic nerve and endocrine function, leading to extensive changes in many aspects of the body system (3). Numerous evidence show that sleep disorders increase the risk of obesity, hypertension, diabetes, dementia, and cardiovascular disease (4–9).

Dry eye disease (DED) is a multifactorial disease of the ocular surface characterized by a loss of tear film homeostasis, causing tear film instability and hyperosmolarity, as well as ocular surface inflammation and damage, and neurosensory abnormalities play etiological roles (10). The incidence of DED was estimated to range from 5 to 50% worldwide (11). DED causes a variety of ocular surface discomfort (such as pain, foreign body sensation, irritation, and photophobia) and visual dysfunction, which not only impedes daily activities, but also exerts psychological impacts (12–14).

Previous studies have found that aging, female sex, systemic diseases (hypertension, diabetes, thyroid disease, anxiety, depression, and so on) are associated with DED development (11, 12, 15, 16). The association between DED and sleep disorders has recently attracted attention (11, 17, 18). It was found that DED patients sleep poorly, and patients with sleep disorders are more likely to have DED (17, 19–28). Some studies were conducted in community populations or cohort populations by collecting questionnaires on sleep and dry eye symptoms (19, 20, 26, 27), however these studies may have drawbacks of selection bias and recall bias. There are also studies suggesting that sedative antidepressants would promote dry eye symptoms and signs (17, 29–31). In the current study, we would like to compare the incidence of DED that developed in participants with or without sleep disorders, using the integrated health care network system data, and evaluate whether sleep disorders was a risk factor for DED.

## Materials and Methods

### Study Population

Yinzhou District, located in the Yangtze River Delta, is one of the core areas of Ningbo. According to the statistical yearbook of Yinzhou District, by the end of 2018, there were 1.34 million permanent residents in Yinzhou District. Data on hospital visits were obtained from the Yinzhou Health Information System (YHIS), which seamlessly links the health information archives, chronic disease registration system, resident death registration system and hospital outpatient system of permanent residents in Yinzhou District, and can provide real-time treatment information of patients in 2 general hospitals, 3 specialized hospitals, 24 community health service centers and 248 community service stations in the whole district. The health information of each resident living in Yinzhou is integrated into YHIS. Detailed information about this system has been described in the previous research (32).

Medical information was classified according to the ICD-10 (International Classification of Diseases, 10th Revision). In this study, we first excluded all patients who were deceased, we selected hospital visits for sleep disorders (ICD-10: G47) and DED (ICD-10: H04.103, H11.104, H16.202 and H18.803) during 2013–2020. We excluded patients diagnosed with DED before sleep disorders. Finally, we identified 128,966 patients with sleep disorders between 2013 and 2020. To design the matched cohort study using the sleep disorder patients as the case group, we established the control group by propensity score matching by adjusting for same sex, same age range and same date of diagnosis for sleep disorders. We used a 1:1 matching method to select the control group (128,966 patients) who were not diagnosed with sleep disorders. Finally, the data used in this study included 257,932 patients from 2013 to 2020.

### Variables

The outcome variable of this study is whether DED will occur after patients were diagnosed with sleep disorders. If diagnosis of DED (ICD-10: H04.103, H11.104, H16.202, and H18.803), it was defined as DED diagnosis, and classified the patient as the DED diagnosis group. In this study, sleep disorders were defined as organic sleep disorders (ICD-10: G47), including insomnia, hypersomnia, narcolepsy/cataplexy, sleep apnea, disorders of the sleep-wake schedule and other sleep disorders. In the study, we included the following independent variables: gender, age, medical history (hypertension, diabetes, thyroid disease, anxiety, depression, heart disease, and arthritis), and medication history of sleep disorders. The ages were classified at 10-year intervals: under 29 years, 30–39 years, 40–49 years, 50–59 years, 60–69 years, over 70 years. At present, the clinical drugs for insomnia mainly include benzodiazepine receptor agonists (BZRAs), melatonin receptor agonists, orexin receptor antagonists, antidepressants and etc. (33, 34). We studied the using condition of sleep medicine in patients with sleep disorders, excluding the drugs mentioned in the guide but not found in the YHIS. Finally, we mainly included BZRAs (estazolam, diazepam, alprazolam, lorazepam), antidepressants (trazodone, mirtazapine, doxepin), and other sedatives (gabapentin, olanzapine).

### Statistical Analysis

In order to study the relationship between DED and sleep disorders, we first determined the frequency and percentage of people with DED diagnosis. Next, we performed a log rank test and generated a survival curve to compare the diagnostic rate of DED between patients with and without sleep disorders. Finally, we performed survival analysis using the univariate cox regression analysis, and then variables with *P* < 0.20 in univariate regression analysis were included in multivariate cox regression analysis to determine the association between sleep disorders and DED, and studied the association of other independent variables with DED. All statistical analyses were performed using SAS statistical software version 9.2 (SAS company, Cary, North Carolina, USA). A *P* < 0.05 was considered to be statistically significant.

## Results

Using the 1:1 propensity score matching method (patients with sleep disorder vs. patients without sleep disorders), we analyzed 257,932 patients with or without sleep disorders between 2013 and 2020. Supplemental Table 1 shows the distribution of the study population for each variable and the 8-year incidence of DED among different variable subgroups. The 8-year incidence of DED was 50.66 and 16.48% in participants with and without sleep disorders, respectively. About 33.57% of patients in our study were diagnosed with DED over 8 years, and incidence was higher in patients with sleep disorders (with sleep disorders: 50.66%, without sleep disorders: 16.48%).

**Figure 1:**
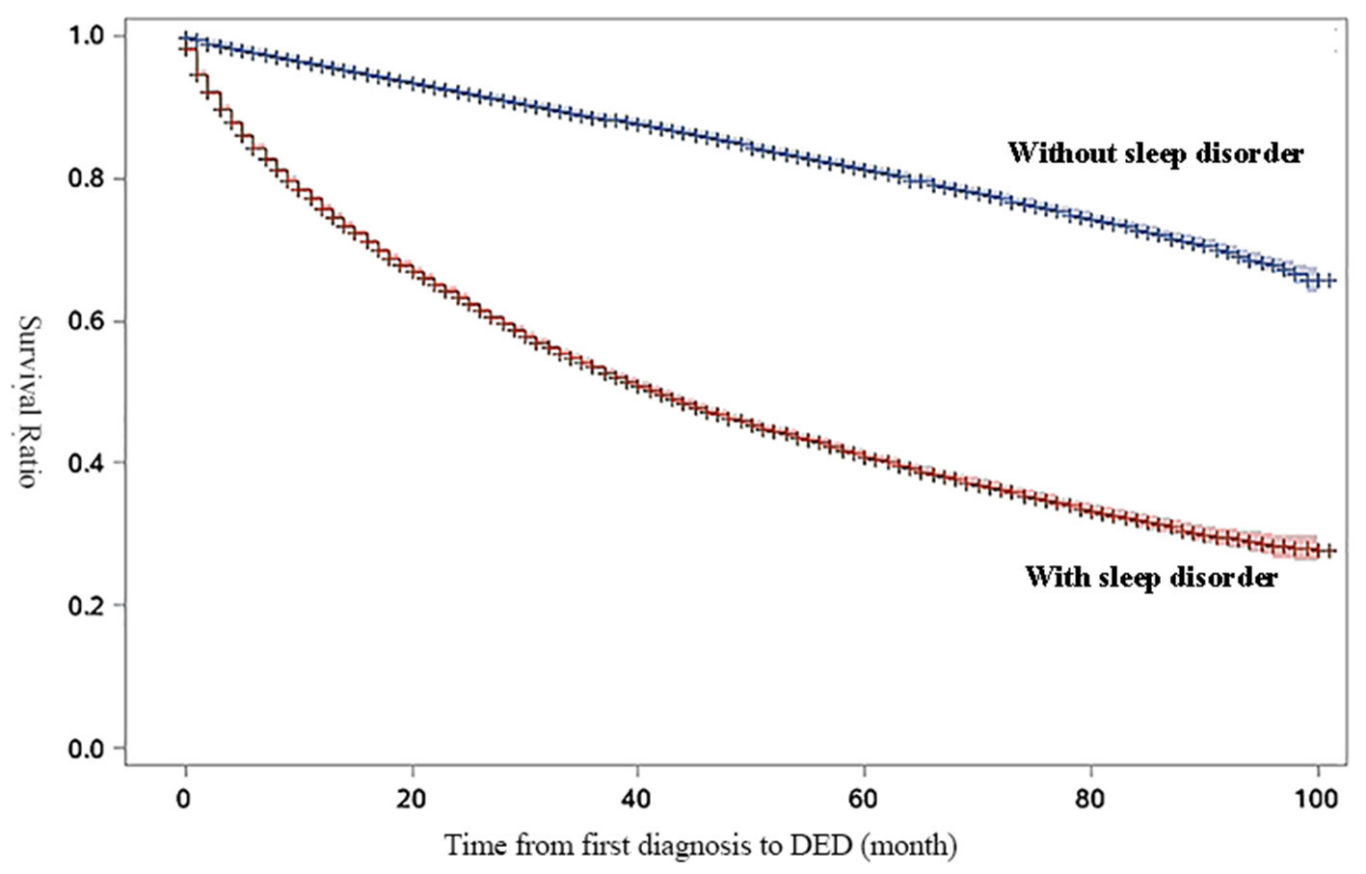
Survival curve for survival-ratio of dry eye disease (DED). The results of log-rank test for time to DED by diagnosis of sleep disorders were statistically significant (HR: 3.81, 95%CI:3.75–3.87, *P* < 0.01).

Figure 1 shows the survival curve to compare the survival rate of DED in patients with sleep disorders and without sleep disorders during the study period. In the survival curve, patients with sleep disorders were more likely to be diagnosed with DED than patients without sleep disorders (*P* < 0.05 for log rank test).

Table 1 shows the results of survival analysis using the Cox proportional hazard model to determine the association between DED and sleep disorders after adjusting other independent variables. Univariate regression analysis showed that sleep disorders, age, gender and coexisting systemic diseases (hypertension, hyperlipidemia, heart disease, and arthritis) were significantly associated with the development of DED (all *P* < 0.05). Then we included variables with *P* < 0.20 from the model 1 for multivariate cox regression analysis. By the multivariate regression analysis, sleep disorders were positively associated with the diagnosis of DED (HR: 3.06, 95% CI: 2.99–3.13, *P* < 0.01), with a three-time increased risk. Among the independent variables, the risk of diagnosing DED was higher in women than in men (HR: 1.25, 95% CI: 1. 22–1.29, *P* < 0.01). Compared with young people who were <30 years, increased age was associated with DED diagnosis (*P* < 0.01), and the risk was the highest in the population aged 40–49 years (HR: 2.04, 95% CI: 1.92–2.18, *P* < 0.01). In addition, only heart disease was positively associated with the diagnosis of DED (*P* < 0.01). At the same time, other coexisting systematic diseases did not have significant association with the diagnosis of DED (*P* > 0.05).

**Table 1:** Results of survival analysis using Cox regression analysis for association between sleep disorders and dry eye disease (DED) in the study population (*n* = 257,932).

**Variables**	**Model 1**		**Model 2**		**Model 3**	
	**HR (95%CI)**	***P***	**HR (95%CI)**	***P***	**HR (95%CI)**	***P***
Sleep disorder	3.81 (3.75, 3.87)	<0.01	3.28 (3.23, 3.34)	<0.01	3.06 (2.99, 3.13)	<0.01
**Sex**
Male	1.00 (-)	-	-	-	1.00 (-)	-
Female	1.20 (1.17, 1.23)	<0.01	-	-	1.25 (1.22, 1.29)	<0.01
Age (years)
<30	1.0 (-)	-	-	-	1.0 (-)	-
30–39	1.86 (1.71, 2.04)	<0.01	-	-	1.94 (1.77, 2.12)	<0.01
40–49	2.00 (1.88, 2.13)	<0.01	-	-	2.04 (1.92, 2.18)	<0.01
50–59	1.71 (1.62, 1.82)	<0.01	-	-	1.73 (1.63, 1.84)	<0.01
60–69	1.44 (1.36, 1.53)	<0.01	-	-	1.46 (1.38, 1.55)	<0.01
≥70	1.18 (1.11, 1.26)	<0.01	-	-	1.18 (1.11, 1.26)	<0.01
**Medical history**
Hypertension	1.12 (1.17, 1.23)	<0.01	1.04 (0.98, 1.10)	0.21	1.02 (0.96, 1.08)	0.51
Diabetes	1.07 (0.98, 1.16)	0.12	0.96 (0.88, 1.04)	0.33	0.95 (0.87, 1.03)	0.19
Hyperlipidemia	1.22 (1.12, 1.34)	<0.01	1.10 (1.01, 1.20)	0.04	1.09 (0.99, 1.19)	0.07
Thyroid disease	0.91 (0.70, 1.17)	0.44	0.85 (0.66, 1.10)	0.21	-	-
Anxiety	1.13 (0.76, 1.69)	0.54	0.98 (0.65, 1.46)	0.90	-	-
Depression	1.19 (0.91, 1.56)	0.19	1.15 (0.88, 1.50)	0.31	1.14 (0.87, 1.50)	0.33
Heart disease	1.38 (1.22, 1.56)	<0.01	1.20 (1.07, 1.36)	<0.01	1.18 (1.05, 1.34)	<0.01
Arthritis	1.39 (1.01,1.93)	0.04	1.23 (0.89, 1.70)	0.21	1.23 (0.89, 1.70)	0.21

*HR , Hazard ratio; CI, Confidence interval*.

*Model 1: Univariate cox regression analysis, and no variables were adjusted*.

*Model 2: Sex and age were adjusted*.

*Model 3: Variables with P <0.20 in univariate cox regression analysis were included for multivariate cox regression analysis*.

**Figure 2:**
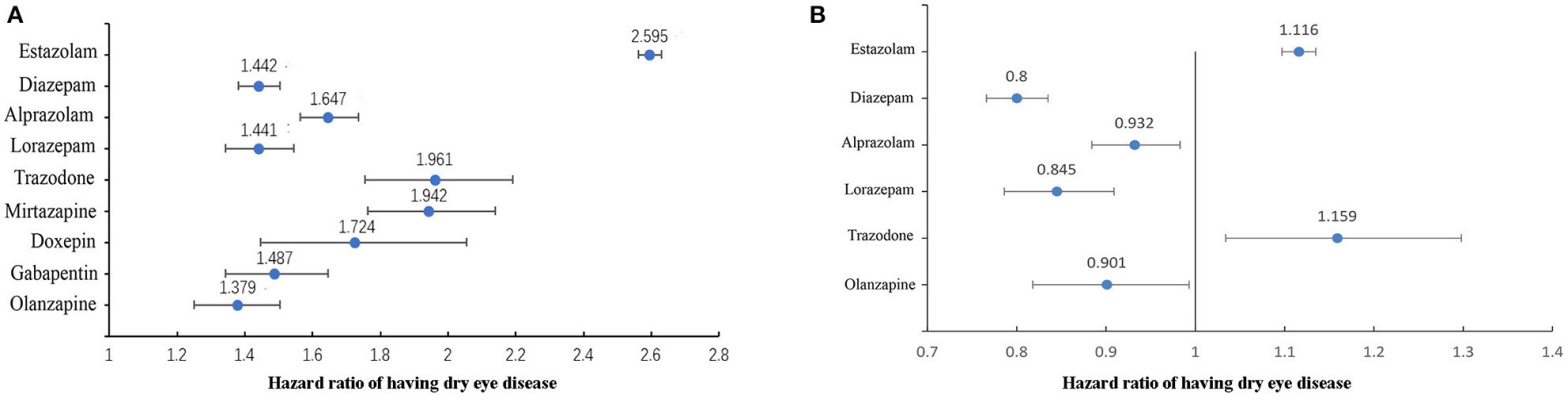
The association between sleep medicine and DED in general population (*n* = 257,932). **(A)** Results of univariate cox regression analysis, all drugs in the figure were statistically significant (*P* < 0.01). **(B)** Results of multivariate cox regression analysis, all drugs in the figure were statistically significant (*P* < 0.05).

Next, we analyzed the effect of sleep drugs on DED in the general population. Figure 2A shows the results of univariate cox regression analysis, all sleep medicine increase the risk of DED (all *P* < 0.01). The sleep medicine with statistical significance (*P* < 0.05) in multivariate cox regression analysis are shown in Figure 2B. It can be seen that estazolam and trazodone were positively associated with the DED.

**Figure 3:**
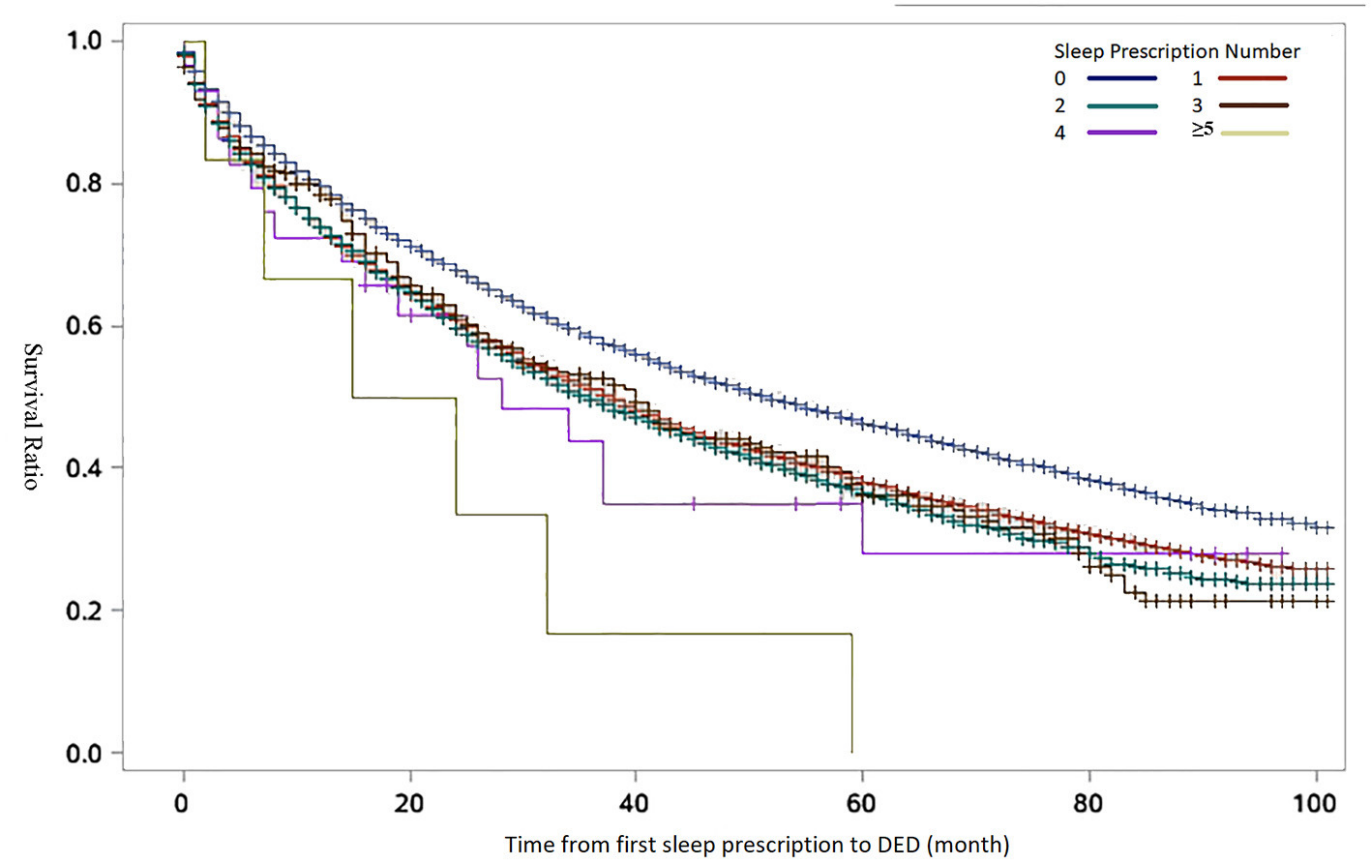
Kaplan-Meier curves for survival-ratio of dry eye disease (DED) over time by the number of sleep prescriptions. The results of log-rank test for time to DED by sleep prescriptions number were statistically significant (*P* < 0.01). The number of sleep prescription refers to the cumulative number of sleep drugs used by the patient.

Additionally, we analyzed the using condition of sleep medicine and its association with DED in sleep disorders patients. Table 2 shows the distribution of the sleep disorders population for each sleep medicine with the Chi square test. The number of cases in Table 2 refers to the person who uses this sleep drug, regardless of whether they use other type sleep drugs or not. Among the sleep disorders, the frequency of estazolam was 63.23%, which was most common in the patients. Compared with patients not using sleep medicine, the incidence of DED significantly increased in the sleep medicine using group (all *P* < 0.01). Figure 3 shows that the incidence of DED over time raised with the increasing of the sleep prescription number used in the sleep disorders.

**Table 2:** The distribution of the sleep disorders population (*n* = 128,966) for each sleep medicine.

**Medicine**	**Total**		**DED**		**P-value**
	***N***	**%**	***N***	**%**	
Estazolam					<0.01
Yes	81,539	63.23	44,116	54.10	
No	47,427	36.77	21,222	44.75	
Diazepam					<0.01
Yes	3,626	2.81	2,188	60.34	
No	125,340	97.19	63,150	50.38	
Alprazolam					<0.01
Yes	2,346	1.82	1,429	60.91	
No	126,620	98.18	63,909	50.47	
Lorazepam					<0.01
Yes	1,288	1.00	775	60.17	
No	127,678	99.00	64,563	50.57	
Trazodone					<0.01
Yes	505	0.39	311	61.58	
No	128,461	99.61	65,027	50.62	
Mirtazapine					<0.01
Yes	616	0.48	402	65.26	
No	128,350	99.52	64,936	50.59	
Doxepin					<0.01
Yes	174	0.13	124	71.26	
No	128,792	99.87	65,214	50.64	
Gabapentin					<0.01
Yes	502	0.39	293	58.37	
No	128,464	99.61	65,045	50.63	
Olanzapine					<0.01
Yes	754	0.58	429	56.90	
No	128,212	99.42	64,909	50.63	

*The proportion of DED for different sleep medicine in sleep disorders population (n = 128,966)*.

*DED, Dry eye disease*.

^*^Chi square test was used to compare the incidence of DED with the use of individual drug.

Similarly, we performed Cox proportional hazard model in the sleep disorder group to determine the relationship between the independent variable and DED. The results of regression analysis are presented in the Table 3. In the model 3 (variables with *P* < 0.20 in univariate cox analysis were included for multivariate cox analysis), the variables of female and 30–39 years significantly increased the DED diagnosis (*P* < 0.01) in the sleep disorders patients. However, the results comparing anxiety with DED were not statistically significant (*P* = 0.08). As is shown in Table 3, the estazolam and trazodone were positively associated with the DED (*P* < 0.05). Contrarily, diazepam and lorazepam showed negative correlation with DED (*P* < 0.05).

**Table 3:** Results of survival analysis using Cox proportional hazard model for association between independent variables and dry eye disease (DED) in the sleep disorder patients (*n* = 128,966).

**Variables**	**Model 1**		**Model 2**		**Model 3**	
	**HR (95%CI)**	***P***	**HR (95%CI)**	***P***	**HR (95%CI)**	***P***
**Sex**
Male	1.00 (-)	-	-	-	1.00 (-)	-
Female	1.14 (1.12, 1.16)	<0.01	-	-	1.15 (1.13, 1.17)	<0.01
**Age (years)**
<30	1.00 (-)	-	-	-	1.00 (-)	-
30–39	1.84 (1.74, 1.95)	<0.01	-	-	1.70 (1.60, 1.81)	<0.01
40–49	1.80 (1.70, 1.90)	<0.01	-	-	1.67 (1.57, 1.77)	<0.01
50–59	1.51 (1.42, 1.60)	<0.01	-	-	1.42 (1.33, 1.50)	<0.01
60–69	1.32 (1.25, 1.40)	<0.01	-	-	1.28 (1.21, 1.36)	<0.01
≥70	1.23 (1.15, 1.31)	<0.01	-	-	1.20 (1.13, 1.28)	<0.01
**Medical history**
Hypertension	1.10 (1.08, 1.12)	<0.01	0.94 (0.93, 0.96)	<0.01	0.87 (0.86, 0.89)	<0.01
Diabetes	1.07 (1.05, 1.09)	<0.01	0.99 (0.97, 1.01)	0.32	0.97 (0.95, 0.99)	<0.01
Hyperlipidemia	1.22 (1.20, 1.24)	<0.01	1.13 (1.11, 1.15)	<0.01	1.11 (1.09, 1.13)	<0.01
Thyroid disease	1.10 (1.04, 1.17)	<0.01	1.12 (1.05, 1.19)	<0.01	1.10 (1.04, 1.17)	<0.01
Anxiety	1.09 (1.08, 1.11)	<0.01	1.04 (1.02, 1.06)	<0.01	0.98 (0.96, 1.00)	0.08
Depression	1.18 (1.15, 1.20)	<0.01	1.15 (1.13, 1.18)	<0.01	1.13 (1.10, 1.16)	<0.01
Heart disease	1.27 (1.25, 1.29)	<0.01	1.16 (1.15, 1.18)	<0.01	1.15 (1.13, 1.17)	<0.01
Arthritis	1.18 (1.14, 1.23)	<0.01	1.15 (1.10, 1.20)	<0.01	1.12 (1.08, 1.17)	<0.01
**Sleep medicines**
Estazolam	1.21 (1.19, 1.23)	<0.01	1.12 (1.10, 1.20)	<0.01	1.12 (1.10, 1.14)	<0.01
Diazepam	0.87 (0.83, 0.90)	<0.01	0.84 (0.81,0.88)	<0.01	0.80 (0.77, 0.83)	<0.01
Alprazolam	1.01 (0.96, 1.06)	0.80	0.98 (0.93, 1.03)	0.36	-	*-*
Lorazepam	0.94 (0.87, 1.00)	0.06	0.92 (0.85, 0.98)	<0.01	0.83 (0.78, 0.90)	<0.01
Trazodone	1.26 (1.12, 1.40)	<0.01	1.25 (1.11, 1.40)	<0.01	1.15 (1.03, 1.29)	0.02
Mirtazapine	1.20 (1.09, 1.33)	<0.01	1.18 (1.06, 1.30)	<0.01	1.09 (0.99, 1.21)	0.09
Doxepin	1.04 (0.87, 1.24)	0.65	0.96 (0.80, 1.15)	0.66	-	*-*
Gabapentin	1.12 (1.05, 1.30)	0.27	1.09 (0.97, 1.22)	0.12	-	*-*
Olanzapine	0.98 (0.89, 1.08)	0.72	0.96 (0.88, 1.06)	0.44	-	*-*

*HR, Hazard ratio; CI, Confidence interval*.

*Model 1: Univariate cox regression analysis, and no variables were adjusted*.

*Model 2: Sex and age adjusted*.

*Model 3: Variables with P <0.20 in univariate cox analysis were included for multivariate cox analysis*.

In the same way, we analyzed the relationship between the independent variable and DED in the patients without sleep disorder by performing Cox proportional hazard model. The results of regression analysis are presented in the Table 4. We can see the variables of female and 40–49 years significantly increased the DED diagnosis (P <0.01) when using multivariate cox analysis. Among the coexisting systemic diseases, only heart disease was significantly correlated with the incidence of DED (*P* = 0.01). Table 4 showed the estazolam, diazepam and gabapentin were all positively associated with the DED (*P* < 0.05).

**Table 4:** Results of survival analysis using Cox proportional hazard model for association between independent variables and dry eye disease (DED) in the patients without sleep disorder (*n* = 128,966).

**Variables**	**Model 1**		**Model 2**		**Model 3**	
	**HR (95%CI)**	***P***	**HR (95%CI)**	***P***	**HR (95%CI)**	***P***
**Sex**
Male	1.00 (-)	-	-	-	1.00 (-)	-
Female	1.20 (1.17, 1.23)	<0.01	-	-	1.25 (1.22, 1.29)	<0.01
**Age (years)**
<30	1.00 (-)	-	-	-	1.00 (-)	-
30–39	1.86 (1.71, 2.04)	<0.01	-	-	1.92 (1.75, 2.10)	<0.01
40–49	2.00 (1.88, 2,13)	<0.01	-	-	2.03 (1.90, 2.16)	<0.01
50–59	1.71 (1.62, 1.82)	<0.01	-	-	1.72 (1.62, 1.83)	<0.01
60–69	1.44 (1.36, 1.53)	<0.01	-	-	1.46 (1.38, 1.54)	<0.01
≥70	1.18 (1.11, 1.26)	<0.01	-	-	1.18 (1.11, 1.26)	<0.01
**Medical history**
Hypertension	1.13 (1.07, 1.20)	<0.01	1.04 (0.98, 1.10)	0.21	1.02 (0.96, 1.08)	0.58
Diabetes	1.07 (0.98, 1.16)	0.12	0.96 (0.88, 1.04)	0.33	0.95 (0.87, 1.03)	0.18
Hyperlipidemia	1.22 (1.12, 1.34)	<0.01	1.10 (1.01, 1.20)	0.04	1.09 (1.00, 1.20)	0.06
Thyroid disease	0.91 (0.70, 1.17)	0.44	0.85 (0.66, 1.10)	0.21	-	-
Anxiety	1.13 (0.76, 1.69)	0.54	0.98 (0.65, 1.46)	0.90	-	-
Depression	1.19 (0.91, 1.56)	0.19	1.15 (0.88, 1.50)	0.31	1.11 (0.85, 1.45)	0.45
Heart disease	1.38 (1.22, 1.56)	<0.01	1.20 (1.07, 1.36)	<0.01	1.17 (1.04, 1.33)	0.01
Arthritis	1.39 (1.01, 1.93)	0.04	1.23 (0.89, 1.70)	0.21	1.21 (0.88, 1.67)	0.25
**Sleep medicines**
Estazolam	1.59 (1.43, 1.77)	<0.01	1.44 (1.29, 1.60)	<0.01	1.42 (1.27, 1.59)	<0.01
Diazepam	2.01 (1.49, 2.72)	<0.01	1.76 (1.30, 2.38)	<0.01	1.69 (1.24, 2.28)	<0.01
Alprazolam	0.82 (0.44, 1.53)	0.54	0.74 (0.40, 1.38)	0.34	-	*-*
Lorazepam	1.14 (0.82, 1.58)	0.44	1.01 (0.73, 1.41)	0.95	-	-
Trazodone	0.83 (0.35, 1.99)	0.68	0.82 (0.34, 1.97)	0.66	-	-
Mirtazapine	1.11 (0.46, 2.66)	0.82	1.25 (0.52, 2.99)	0.62	-	-
Doxepin	2.31 (0.33, 16.33)	0.40	1.85 (0.26, 13.08)	0.54	-	*-*
Gabapentin	1.75 (1.41, 2.17)	<0.01	1.51 (1.22, 1.88)	<0.01	1.51 (1.22,1.88)	<0.01
Olanzapine	0.78 (0.53, 1.14)	0.20	0.75 (0.51, 1.10)	0.14	-	*-*

*HR, Hazard ratio; CI, Confidence interval*.

*Model 1: Univariate cox regression analysis, and no variables were adjusted*.

*Model 2: Sex and age adjusted*.

*Model 3: Variables with P <0.20 in univariate cox analysis were included for multivariate cox analysis*.

## Discussion

In the current study, we found that sleep disorder is a risk factor of DED, with a three-time increased risk to the subjects without sleep disorders. Besides, the use of estazolam increased the risk of DED regardless of the presence of sleep disorder.

In our study, the 8-year incidence of DED in the study population was 33.57%. Many previous population-based studies have indicated a correlation between sleep disorders and DED (19, 20, 24–27, 35). Han et.al reported that sleep disorders were related to dry eye incidence rate (HR:1.32), and sleep had a greater impact on DED in male and young patients (36). In a nationally population-based survey in Korea, people with sleep duration shorter than 5 h were found to be 20% more likely to suffer from DED, compared to those with more than 6 h of sleep (35).Hanyuda et al. also showed that sleep deprivation and poor sleep quality were significantly related to DED in a Japanese population (26). Additionally, two large studies conducted in Hangzhou and Singapore also revealed that poor sleep quality and short sleep time were significantly correlated with DED (19, 20). Our results are similar with the above studies, compared with patients without sleep disorders, patients with sleep disorders have a higher risk of DED (HR:3.06).

The association between sleep disorders and DED are based on physiological findings. Sleep disorders are often related to autonomic nerve dysfunction, which could affect the parasympathetic fibers in the lacrimal gland, resulting in reduced tear secretion (37, 38). Sleep deprivation may also inhibit peroxisome proliferator-activated receptor alpha (PPARα) signaling which increase oxidative stress leading to DED (39). In addition, the activation of the hypothalamic–pituitary–adrenal axis during sleep may lead to a relatively dehydrated state, thereby reducing tear secretion (40, 41).

Our results also found that use of sleep medications in the sleep disorders was significantly associated with DED. Kawashima et al. have demonstrated that sedative antidepressants could significantly decreased tear break-up time of DED patients (29). Antidepressants could promote dry eye symptoms through anticholinergic side effects (inhibition of cholinergic nerve fibers in meibomian and lacrimal glands) or other potential pharmacological mechanisms to reduce tear secretion (17, 30). Using the multivariate cox regression analysis, we found that there was a significantly positive association between estazolam and the incidence of DED in both sleep disorder and non-sleep disorder participants. Estazolam, a kind of benzodiazepine drugs (BZDs), can non selectively activate different γ subunits on γ-aminobutyric acid receptor A and has pharmacological effects of sedation, hypnosis, antianxiety, muscle relaxation and anti-convulsion (42). Estazolam has side effects of anticholinergic activity (43), which reduces the secretion of tears, resulting in DED. In the present study, the ratio of estazolam use amounts to 63.23% in the subjects with sleep disorders, while the ratio of other sleep medicine were below 3%, which may limit the power of detection small effect. Therefore, the association between other sleep medicines and dry eye needs to be evaluated in a larger study with sufficient sleep medication users.

The advantage of current study lies in the large sample size based on data from an integrated health care network covering a whole district in southeast China. Most of the previous studies used questionnaires (19, 20, 26, 27), which would cause recall bias. Our data from the medical information system can avoid such bias. The Cox proportional hazards model used in the current study is a reliable method to analyze the incidence of dry eye in sleep disorders patients, since it took the impact of time into consideration, which is different from the previous cross-sectional population studies (19, 20, 26, 27). Besides, the paired cohort design using propensity score matching (1:1) increased the reliability of our study. However, limitations are unavoidable, and the main one is the diagnosis of DED and sleep disorder may not be in consistency among the hospitals and community health centers during the 8-year period. However, the paired cohort design made up for this drawback to a certain degree.

In conclusion, our findings suggest that sleep disorders is a potential risk factor for DED and the use of sleep medicines might be associated with dry eye development. Patients with sleep disorders or those using sleep medicines should be monitored for development of dry eye.

## Data Availability Statement

The raw data supporting the conclusions of this article will be made available by the authors, without undue reservation.

## Ethics Statement

The studies involving human participants were reviewed and approved by Institutional Review Board of the affiliated Ningbo Eye Hospital of Wenzhou Medical University. Written informed consent for participation was not required for this study in accordance with the national legislation and the institutional requirements.

## Author Contributions

QZ designed the study. SL, FW, ZL, and KF analyzed the data. YS and JB checked the data. QZ and SL wrote the manuscript. HW revised the manuscript. PS, HL, and WC instructed the whole study and revised the manuscript. All authors contributed to the article and approved the submitted version.

## Funding

This work was financially supported by the Chinese National Key Research and Development Projects(2019YFC0840708).

## Conflict of Interest

The authors declare that the research was conducted in the absence of any commercial or financial relationships that could be construed as a potential conflict of interest.

## Publisher's Note

All claims expressed in this article are solely those of the authors and do not necessarily represent those of their affiliated organizations, or those of the publisher, the editors and the reviewers. Any product that may be evaluated in this article, or claim that may be made by its manufacturer, is not guaranteed or endorsed by the publisher.
