# Supplementary material for: The Association Between Sleep Disorders and Incidence of Dry Eye Disease in Ningbo: Data From an Integrated Health Care Network
Source: Front Med (Lausanne). 2022 Feb 4;9:832851. doi: 10.3389/fmed.2022.832851 (PMC8854755; doi:10.3389/fmed.2022.832851)
Supplement: Supplementary file 1 [file Table_1.DOCX]

| Supplemental table 1. General characteristics of the study population(n=257932). | | | | | | | | | | | | |  |  |
| --- | --- | --- | --- | --- | --- | --- | --- | --- | --- | --- | --- | --- | --- | --- |
| Variables | | Total | | DED | | | | | | | | Non-DED | | |
|  | | N | % | | | | | | N | % | | N | | % |
| Sleep disorder | |  |  | | |  | | | |  | |  | |  |
| Yes | | 128966 | 50.00 | | | | 65338 | | | 50.66 | | 63628 | | 49.34 |
| No | | 128966 | 50.00 | | | | 21260 | | | 16.48 | | 107706 | | 83.52 |
| Sex | |  |  | | |  | | | |  | |  | |  |
| Male | | 97848 | 37.94 | | | | 30545 | | | 31.22 | | 67303 | | 68.78 |
| Female | | 160084 | 62.06 | | | | 56053 | | | 35.01 | | 104031 | | 64.99 |
| Age（years） | |  |  | | |  | | | |  | |  | |  |
| ＜30 | 19226 | 7.45 | | | | 2684 | | | | 13.96 | 16542 | | 86.04 |  |
| 30~39 | 39142 | 15.18 | | | | 7785 | | | | 19.89 | 31357 | | 80.11 |  |
| 40~49 | 57951 | 22.47 | | | | 16407 | | | | 28.31 | 41544 | | 71.69 |  |
| 50~59 | 57705 | 22.37 | | | | 21124 | | | | 36.61 | 36581 | | 63.39 |  |
| 60~69 | 49656 | 19.25 | | | | 22169 | | | | 44.65 | 27487 | | 55.35 |  |
| ≥70 | 34252 | 13.28 | | | | 16429 | | | | 47.97 | 17823 | | 52.03 |  |
| Medical history | |  |  | | |  | | | |  | |  | |  |
| Hypertension | |  |  | | |  | | | |  | |  | |  |
| Yes | | 97813 | 37.92 | | \| 50050 \| \| --- \| | | | | | 51.17 | | 47763 | | 48.83 |
| No | | 160119 | 62.08 | | | 36548 | | | | 22.83 | | 123571 | | 77.17 |
| Diabetes | |  |  | | |  | | | |  | |  | |  |
| Yes | | 34363 | 13.32 | | | 17524 | | | | 51.00 | | 16839 | | 49.00 |
| No | | 223569 | 86.68 | | \| 69074 \| \| --- \| | | | | | 30.90 | | 154495 | | 69.10 |
| Hyperlipidemia | |  |  | | |  | | | |  | |  | |  |
| Yes | | 74186 | 28.76 | | | 40977 | | | | 55.24 | | 33209 | | 44.76 |
| No | | 183746 | 71.24 | | | 45621 | | | | 24.83 | | 138125 | | 75.17 |
| Thyroid disease | | |  | | |  | | | |  | |  | |  |
| Yes | | 2111 | 0.82 | | | 1082 | | | | 51.26 | | 1029 | | 48.74 |
| No | | 255821 | 99.18 | | | 85516 | | | | 33.43 | | 170305 | | 66.57 |
| Anxiety | |  |  | | |  | | | |  | |  | |  |
| Yes | | 27549 | 10.68 | | | 16186 | | | | 58.75 | | 11363 | | 41.25 |
| No | | 230383 | 89.32 | | | 70412 | | | | 30.56 | | 159971 | | 69.44 |
| Depression | |  |  | | |  | | | |  | |  | |  |
| Yes | | 14874 | 5.77 | | | 9079 | | | | 61.04 | | 5795 | | 38.96 |
| No | | 243058 | 94.23 | | | 77519 | | | | 31.89 | | 165539 | | 68.11 |
| Heart disease | |  |  | | |  | | | |  | |  | |  |
| Yes | | 55466 | 21.50 | | | 31485 | | | | 56.76 | | 23981 | | 43.24 |
| No | | 202466 | 78.50 | | | 55113 | | | | 27.22 | | 147353 | | 72.78 |
| Arthritis | |  |  | | |  | | | |  | |  | |  |
| Yes | | 4148 | 1.61 | | | | | 2423 | | 58.41 | | 1725 | | 41.59 |
| No | | 253784 | 98.39 | | | | | 84175 | | 33.17 | | 169609 | | 66.83 |
| DED=dry eye disease. | |  |  | | | | |  | |  | |  | |  |
